# A 3-arm randomized controlled trial on the effects of dance movement intervention and exercises on elderly with early dementia

**DOI:** 10.1186/s12877-015-0123-z

**Published:** 2015-10-19

**Authors:** Rainbow Tin Hung Ho, Jacob Kai Ki Cheung, Wai Chi Chan, Irene Kit Man Cheung, Linda Chiu Wah Lam

**Affiliations:** Centre on Behavioral Health, The University of Hong Kong, 2/F, The Hong Kong Jockey Club Building for Interdisciplinary Research, 5 Sassoon Road, Pokfulam, Hong Kong, China; Department of Social Work and Social Administration, The University of Hong Kong, Room 534, Jockey Club Tower, Centennial Campus, Hong Kong, China; Sau Po Centre on Aging, The University of Hong Kong, 2/F, The Hong Kong Jockey Club Building for Interdisciplinary Research, 5 Sassoon Road, Pokfulam, Hong Kong, China; Department of Psychiatry, The University of Hong Kong, 2/F, New Clinical Building, Queen Mary Hospital, 102 Pokfulam Road, Hong Kong, China; Department of Psychiatry, The Chinese University of Hong Kong, Tai Po Hospital, G/F Multicentre, Tai Po, Hong Kong, China

**Keywords:** Dance movement, Dementia, Elderly, Exercise, Chinese, Randomized controlled trial, Salivary cortisol

## Abstract

**Background:**

Dementia is characterized by a progressive decline and deterioration of brain regions such as memory, spatial navigation and language, along with disturbances in daily functioning. Non-pharmacological interventions that offer a holistic approach by targeting cognitive functioning, prognosis and the psychological and social effects of dementia require rigorous investigation. The well-established benefits of physical activity for cognitive functioning and psychological support in dementia have been observed with dance-movement intervention. There is substantial evidence that dance-movement interventions provide emotional and social advantages. Thus, a randomized controlled trial (RCT) is planned to investigate the positive effects of a dance movement intervention, compared with mild physical exercise, on the physical and psychological well-being of elderly Chinese individuals with early dementia.

**Methods/Design:**

A 3-arm RCT with waitlist control design will be used in this study. Two hundred and one elderly participants with very mild to mild dementia will be screened and randomized into the following groups: (i) dance movement based intervention, (ii) stretching and exercise intervention and (iii) no intervention waitlist-control group. The two intervention groups will receive a 1-h intervention, twice a week, for 12 weeks. The participants will be assessed four times over the course of 12 months: baseline before randomization, post-intervention (3 months), 6 months from baseline and 12 months from baseline. The primary outcomes will be compared between assessment points and between groups on neuropsychiatric symptoms, psychosocial well-being and cognitive and daily functioning. Secondary outcomes will assess the changes in salivary cortisol levels and their relationships with the primary outcome measures.

**Discussion:**

This study will provide substantial evidence of the efficacy of a dance-movement-based intervention in slowing down dementia progression, due to its ability to act as a buffer against decline and improve areas affected by dementia. We also anticipate an association between cortisol levels and the outcome measures. The further development of this intervention into a structural program may be warranted for early psychosocial support among elderly populations.

**Trial registration:**

The trial has been registered in the Chinese Clinical Trial Registry (ChiCTR-IOR-15006541).

## Background

Dementia is a progressive neurodegenerative condition marked by cognitive impairments in memory, language, recognition and movement, which interfere with daily and social activities and impair all aspects of well-being. Pathologically, there is a shrinking of the brain regions involved in memory, spatial navigation and language [1]. In addition to significant cognitive deterioration, damage to the amygdala negatively affects emotions and behavior [2]. The progressive decline in daily functioning (with losses in communication, memory and social roles), difficulty expressing feelings and the certainty of further deterioration may lead to anxiety [3, 4]. Disruptive behavior, poor self-care and additional financial burdens result in stress for caregivers in addition to other behavioral and psychological manifestations. Thus, dementia can be agonizing for both patients and caregivers [5, 6]. Locally in Hong Kong, 18 % of elderly individuals over the age of 70 suffer from very mild or mild dementia, and 50–70 % of dementia patients exhibit disruptive behavior or psychiatric symptoms such as sleep disturbance, irritability, depression and aberrant motor behavior [6, 7].

**Fig. 1:**
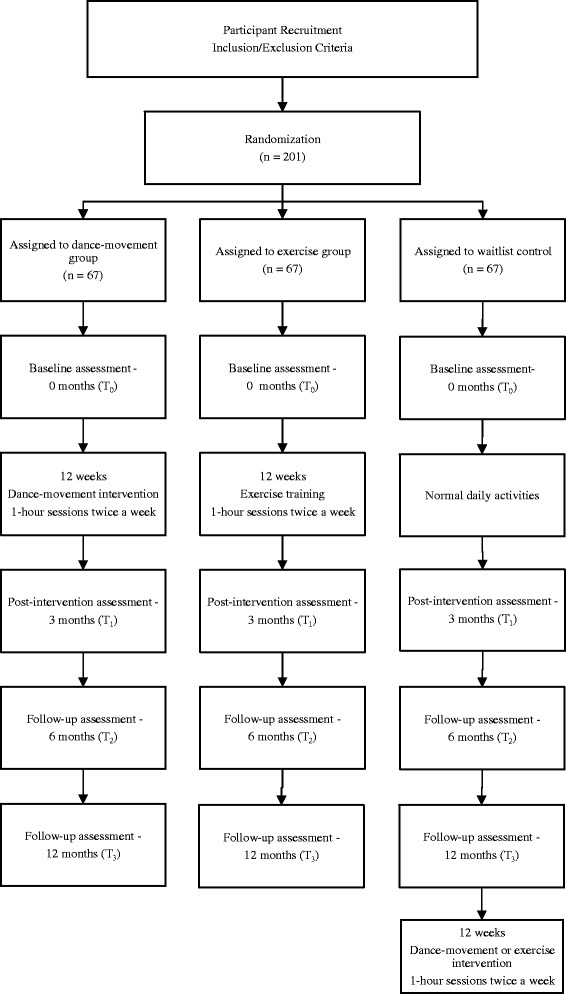
Flow diagram of the progress through the phases of the 3-arm randomized trial of enrollment, intervention, allocation and data collection points. Research design is illustrated by showing the treatment duration to be received by each intervention group, the waitlist control condition received by the control group and the study’s data collection points throughout the whole study

Despite how negative emotions can influence cognitive deterioration, patients’ psychosocial well-being is sometimes neglected and regarded as secondary to managing deteriorating cognitive functioning, which has resulted in a lack of research on the non-cognitive manifestations of dementia [8]. Interventions that target patients’ physical and mental well-being have a larger capacity to effectively improve the multifaceted impairments generated by dementia than physical well-being alone [9]. Not only is the efficacy of medical treatment for dementia limited, with modest clinical influence and problematic side-effects [10, 11], but studies measuring the antipsychotics prescribed for behavioral and psychological symptoms have been reported to increase mortality [12, 13].

### Effectiveness of physical activity on dementia

McLaren reviewed interventions for dementia involving aerobic, balance, flexibility and resistance exercise and concluded that exercise benefits both functioning and quality of life [10]. More specifically, the systematic review provided clinical trial evidence that non-pharmacologic interventions can delay the progression of functional impairment or disability among dementia patients [10]. One neurobiological study found that the hippocampus, which is responsible for memory and is one of the first areas to degenerate in dementia, increases in volume as both acute and chronic aerobic exercise increases [14]. This indicates that activating the neuro-hormonal mechanisms through exercise can induce cognitive enhancement [14].

Additionally, a controlled study on physical exercises supported by music for moderate to severe dementia patients found improvements in overall cognition, especially information processing and recall [15].

### Benefits of dance movement therapy in dementia treatment

Similarly, dance movement used as a therapeutic intervention combines the physical benefits of exercise with psychosocial therapeutic benefits [16]. Although dance-movement interventions for dementia patients are only mild/moderate forms of physical activity, the level of exertion prompted by a weekly hour of dance among healthy elderly patients was shown to be sufficient to change brain growth factors supporting brain plasticity, similar to the changes induced by exercise [17]. Overall, Foster [17] illustrated that dancing activates the areas in the brain associated with perception, emotions, executive function, memory and motor skills. Specifically, dance and movement improvisation uses frontal lobe resources while movement relies on path integration and visual tracking, which activate the hippocampal and entorhinal networks associated with spatial memory—an area of profound impairment in dementia [17]. In a randomized controlled study using 9 weeks of dance-movement therapy among dementia patients in nursing homes, improved visuospatial abilities were observed and the deterioration of self-care was prevented [18].

The components of dance-movement therapy such as rhythm and tapping target at different cognitive domains. Dance movement affects procedural memory abilities such as motor and cognitive skills, which is highly applicable because motor learning is comparatively well-preserved in dementia [19, 20]. There is growing empirical evidence that moving to rhythm enhances the body’s ability to encode rhythmical movement [21, 22], which is reflected in one’s gait. Certain therapeutic aims such as improving balance among elderly Parkinson’s patients can be achieved by enhancing rhythmical movement—an inherent component of dance [23]. For elderly dementia patients, rhythmic physical activity appears to improve mood and memory [24]. The behavior of elderly residents in nursing care stabilized, with reductions in agitation and wandering upon completion of a psychosocial-focused dance intervention [25]. Emotionally salient and meaningful dance movement can promote both functional and emotional skills and behavior that remain useful to the elderly, even as they progress into the later stages of dementia [26]. Rhythmic movement will be particularly emphasized in the proposed dance-movement intervention for its potential benefit to the elderly.

Psychologically, there is preliminary evidence that dance movement can promote positive changes by facilitating self-expression and communication among elderly dementia patients [25]. More importantly, a non-verbal approach provides a psychotherapeutic medium of expression for patients with dementia when their verbal abilities decline [3]. Group dance, in particular, supports a sense of integration and connection that counteracts the difficulty of orientating oneself in time and space [26]. Additionally, group dance can ease feelings of fear and isolation, as disorientation often leads to a fragmented sense of self [26]. Elderly patients facing gradual memory loss may also benefit from the propensity of dance to evoke reminiscence and restore long-term memories or associated emotions [3].

### Salivary cortisol levels and dementia

Salivary cortisol is a neuroendocrine indicator of hypothalamic-pituitary-adrenal (HPA) axis activity, responsible for stress and immune regulation in the body [27]. An elevated cortisol level has recently been found to correlate with rapid progression of dementia [28]. Prolonged heightening of cortisol levels among the elderly is predictive of reduced hippocampal volume, resulting in poor performance in hippocampus-dependent memory tasks, compared with controls [29]. Abnormal cortisol level patterns have more often been found in moderate dementia, with relatively flat cortisol patterns seen in severe dementia [30, 31]. Elderly individuals who are motivated to strengthen their social ties have exhibited healthier cortisol activity across the day [32]. How healthy cortisol activity induced by moderate exercise extends its effect on dementia symptoms has not been properly investigated. The assessment of cortisol can help to elucidate the interrelationship between immunity, psychological health and dementia-related decline.

Although dance-movement interventions are clinically well-established, evidence remains descriptive and the designs of related studies have limitations [25]. Given the increasing aging population, research on non-pharmacological interventions has remained limited. Consequently, a non-pharmacological intervention adopting a holistic approach to improving the overall physical and mental well-being of people with early dementia should be explored. This study aims to examine the effects of a dance movement based intervention for elderly patients with very mild to mild dementia, compared with an exercise intervention and no intervention control conditions.

### Research objectives

The primary objective of the proposed study is to explore the effects of a dance movement based intervention on dementia-related indicators such as neuropsychiatric symptoms (behavior, psychosis, ,apathy), psychosocial well-being (depression, loneliness and current affective state), physiological stress (indicated by cortisol), cognitive functioning (executive function, episodic memory, visuospatial construction) and daily functioning. The secondary objectives of this study are to explore the proposed intervention’s effect on dementia prognoses and examine how cortisol levels relate to neuropsychiatric, psychosocial, cognitive and functional measures in early dementia. Through these objectives we aim to provide insight into possible mechanisms of change and interactions among the aforesaid variables after dance-movement intervention.

We hypothesize that upon completion of the dance-movement intervention, elderly dementia patients will experience a greater delay in the progression of dementia severity compared with the exercise and control groups. Furthermore, those who participate in the dance-movement intervention should experience greater resilience against dementia-related decline, compared with the exercise and control groups. We expect to find an association between cortisol levels and neuropsychiatric, cognitive and daily functioning, in addition to psychosocial measures.

## Methods/Design

### Research design

This study will adopt a 3-arm RCT design with waitlist control. After screening and collecting baseline data, computer-generated random numbers will be used to randomize the eligible participants into one of three intervention groups: dance movement, exercise or waitlist control. The waitlist control group will be provided with a dance-movement or exercise intervention upon study completion. All of the participants will continue to receive regular medication and care. Four repeated assessments will be conducted for all of the participants at baseline, post intervention (3 months), and 6 and 12 months after the baseline. The manuscript and flow diagram (Fig. 1) adheres to CONSORT guidelines.

### Participant eligibility and recruitment

Eligible elderly participants (aged 65 and above) with a clinical diagnosis of dementia (DSM IV) or major/mild neurocognitive disorder (DSM V) will be recruited from the psychogeriatric outpatient departments of local hospitals, elderly daycare centers and community centers by their treating psychiatrists or facility staff. Before baseline assessment, a Clinical Dementia Rating (CDR) evaluation will be conducted by the team’s psychiatrist and a trained CDR rater to screen for very mild to mild dementia (CDR 0.5–1).

#### Inclusion criteria

- CDR of 0.5–1.
- Chinese patients aged 65 to 90.
- Community dwelling.
- Mobility of at least the upper limbs and body.
- Sufficient visual and auditory abilities to complete assessments.
- Stable doses of medication for at least 30 days before screening.

#### Exclusion criteria

- Concurrent major psychiatric disorder (e.g. major depressive disorder, bipolar disorder, schizophrenia) or drug and alcohol abuse.
- Severe illness or pain that would lead to significant deterioration in health, or that would limit participation in the interventions (e.g. metastatic cancer, musculoskeletal disorders, etc.).

A trained research assistant will obtain informed consent from the participants upon fulfilling the inclusion and exclusion criteria. The eligible and consented participants will then be randomized into one of the three aforementioned treatment conditions.

### Sample size determination

A sample size of 131 has been determined. Due to the presence of multiple independent variables, multiple regression modeling with a medium effect size (f2) of 0.15, at 0.8 power and a significance level of 0.05, was used to calculate the sample size. The experimental, clinical and demographic variables anticipated to affect the intervention outcomes were included in the model. Based on prior local trials with early dementia patients, the recruitment of 201 participants is required (67 per arm), assuming an attrition rate of about 35 % [33].

### Intervention groups

The dance-movement intervention will be led by a registered dance-movement therapist or one in training. The program will be modified from a pre-existing dance/movement therapy program applied within various Chinese populations in the past decade with evidenced benefits. The modified elderly program has also been tested in a group of elderly participants and proved both feasible and well-received. The dance-movement intervention program to be used in the proposed study will include gentle body movements with special emphasis on rhythm and contralateral movement on both sides of the body to enhance coordination and stimulation between right and left cerebral hemispheres [21, 22]. Simple group dances will encourage the participants’ ability to remember steps and sequences, movement games will improve mood and vitality and improvisational dance movement will foster imagination, creativity and personal expression [17, 26]. Movement interactions among group members are expected to enhance communication and social exchanges. Finally, vocal sharing about movement experiences should provide articulation opportunities that may help the participants in building mutual support and care. To cater to the elderly, movements will be conducted both in standing and sitting positions. The structure and schedule of each session will be similar, with the content adjusted to suit the needs and dynamics of the group.

The exercise intervention group will receive a mild to moderate exercise program of comparable length and intensity, conducted by an instructor trained by a qualified fitness coach. Each session of the exercise and dance-movement interventions will include a warm up (15 min), stretching and joint movements (15 min), exercising with towels (15 min) and cool down (15 min). Heart rates will be monitored throughout the entire dance-movement or exercise routine using a portable heart rate monitor to maintain a similar mild to moderate level of exercise exertion in both groups (40–60 % of the VO2 max value). For both the dance-movement and exercise arms, interventions will span 12 weeks, with 1-h sessions twice a week (a total of 2 h per week) in groups of about 6-12 participants each. Based on studies with exercise interventions, a 12-week intervention supports significant result comparison [10]. The waitlist controls will continue with routine care and can join either a dance-movement or exercise intervention after the 12-month assessment.

### Measurements

The assessments will be conducted by a researcher blinded to the randomization, trained in using the instruments. CDRs will be assessed by a psychiatrist and a trained CDR rater blinded to the randomization. Self-rated scales will be completed by the participants under guidance.

1. Screening Instrument:
  1.1 *The CDR* is a semi-structured interview with six dimensions: memory, orientation, judgment, community affairs, hobbies and habits and personal care. Each item from its corresponding dimension will then be converted to an average score for that dimension. The average score for each dimension will be converted to a defined range and a combination of those ranges will produce an overall CDR rating ranging from 0 to 5 [34]. In the selection of very mild to mild grade dementia, participants with scores from 0.5 (and 0.5 or more on 3 or more domains) to 1 will be selected in accordance with previous studies [7]. The CDR will be assessed before randomization and at the final 12-month follow up.
2. Psychosocial Assessments:
  2.1 *The Chinese Neuropsychiatric Inventory (NPI)* assesses 12 psychological and behavioral symptoms of patients with neurodegenerative disorders. Categorized as behavioral problems (agitation/aggressiveness, disinhibition, irritability, aberrant motor behavior), psychosis (delusions and hallucinations), mood disturbance (depression, anxiety, sleep, appetite, apathy) and euphoria. This tool will be administered by a trained research assistant to their main caregivers based on informant ratings [35].
  2.2 *The Abbreviated Chinese Geriatric Depression Scale (ac-GDS)* is a 4-item version selected to provide a simple and reliable assessment of depressive mood in elderly dementia patients. With depression being one of the most common concomitants of dementia, participants’ subjective well-being can be profoundly affected. The GDS is self-rated by the participants [36].
  2.3 *The Chinese de Jong Fierveld Loneliness Scale (c-JFLS)* is a 6-item self-reported scale based on a 3-point scale. The items are associated with feelings of being alone, rejection and a sense of having support [37]. The scores are associated with social variables related to contact with family, relatives and close persons, or just having people around.
  2.4 *The Visual Analogue Mood Scale (VAMS)* is a reliable and sensitive single-item assessment of 8 affective states: afraid, confused, sad, angry, energetic, tired, happy and tense [38]. It was designed to offer a simple and direct means of assessing various mood states in neurologically impaired adults. On a visual analogue scale of 0 to 100, the respondents are asked to rate the intensity of their mood. The presence of positive mood markers is also a hallmark of this scale.
3. Cognitive Functioning:
  Cognitive functioning tests will be assessed by a trained research assistant.
4. *The Digit Span Test* is an indicator of cognitive impairment in dementia to assess executive functioning. The tasks require immediate memory of numbers and the maintenance and manipulation of information in the mind. This test has good validity for elderly participants in Hong Kong [39].
5. *Fuld Object Memory Evaluation* measures episodic memory, which is a profound area of loss in dementia. The tasks require the remembering of ten unrelated items over five immediate recall trials and a delayed recall trial. This evaluation makes use of multiple sensory systems, not just auditory or visual cues, and is thus more pertinent to what is being promoted through dance movement. The Chinese version has been validated [40].
6. *The Trail Making Test (Parts A and B)* measures visuospatial construction as the participant draws lines between alternate numbers, which requires visual search, attention and mental flexibility. Chinese norms for the scale have been established [41].
7. Functioning Assessments
  4.1 *The Lawton Instrumental Activities of Daily Living -Hong Kong version (IADL-hk)* is an 8-item, self-reported measure of community living and functioning skills [42]. Daily functional skills including cooking, use of the telephone, use of transport, household management, money management and shopping skills, measured on a scale of 0 to 8.
8. Biomarker
  5.1 Salivary cortisol collection will be conducted using the “Salivette” collection device, which includes a cotton swab placed under the tongue by the participants, with the assistance of a daycare nurse. Saliva samples will be collected at 5 prescribed times (awakening, 45 min post-awakening, 12:00, 5:00 and 9:00 pm) and kept frozen at −20 °C. Smoking, eating and drinking is not allowed 1 h before saliva collection.
  5.2 Daily condition measures of the participants’ health behavior and activities on the day of saliva collection will be collected along with cortisol readings. Self-rated on a scale of 1 to 10, these conditions include (i) sleep quantity that day (hours of sleep at night and nap hours), (ii) subjective sleep quality rated on a scale of 1 to 10, (iii) smoking habit and the number of cigarettes consumed that day, (iv) alcohol/coffee drinking habit and the approximate intake amount that day and (v) subjective evaluation of stress level on the day of collection. All of the above measures will affect the diurnal cortisol rhythm and must be considered in data interpretation.
9. Socio-demographic and Clinical Information
  6.1 The patients’ socio-demographic and clinical information will be solicited. Socio-demographic data will include age, gender, education level and marital status. The type, frequencies and durations of regular exercise will also be recorded. Clinical information will include the duration and severity of dementia diagnoses, symptoms, other psychiatric diagnoses, other illnesses, medications, other treatments and hospitalization during the study. Their medications and other adjunctive treatments, including psychosocial care, will also be collected to control for confounding variables when modeling outcomes.

### Exploring the efficacy of dance-therapy intervention

An intention-to-treat analysis will be used to prevent participant selection biases attributed by study discontinuation. The efficacy of the dance-movement intervention will be compared to the exercise intervention and waitlist control using a repeated measures analysis of variance across the four assessment time points after adjusting for confounding variables. An ordinary least squares regression will be used to understand the relationships between the dependent variables (outcomes) and independent variables (covariates). To examine the interventions’ effects on prognoses and the likelihood of participants’ progressing to more severe stages of dementia, odds ratios will be conducted on CDR scores. The data analysis procedures will be conducted using SPSS and MPlus software with criteria for levels of significance set at *p* < .05.

### Analysis of salivary cortisol

To obtain cortisol levels from the saliva samples, the samples will be thawed and then centrifuged at 3000 rpm for 15 min at room temperature. The cortisol levels will be determined using an enzyme-linked immunoabsorbent assay kit (EIA, Salimetrics). The assay sensitivity for the kit is 0.007 g/dl and the intra- and inter-assay coefficients of variation are 3 and 10 %, respectively. Due to the skewed distribution of cortisol data, natural logarithm will be used to transform the raw cortisol data and yield a normal distribution for all of the analyses. An average of the five collected samples across that day will produce the mean cortisol level. Diurnal cortisol rhythm will be calculated by linear regression of the log transformed cortisol on collection time, and total cortisol level will be measured by the area under the curve.

### Relationships between cortisol levels and physical and psychological determinants

Correlation analyses will be used to explore the interrelationships between baseline diurnal cortisol patterns and psychosocial, cognitive and functioning measures. Using a variant of multiple regression modeling for the nested structure of our data, a two-level individual growth curve model will be used to explore the individual trajectories of changes in cortisol levels over time, and the complex relationships between different variables. The cortisol measures at the five daily time points will be nested within the participants.

#### Ethics

This proposal has obtained ethical approval from the Institutional Review Board of the University of Hong Kong/Hospital Authority Hong Kong West Cluster (HKU/HA HKW IRB). Institutional Review Board reviewer who approved this proposal was Professor C L Lai, Deputy Chairman and Dr. James Ho, Deputy Chairman of the HKU/HA HKW IRB. Informed written consent will be obtained from the participants and codes will replace names to ensure privacy. The data will be locked up and destroyed 3 years after the study ends.

## Discussion

Although the benefits of physical exercise have been well documented, how a dance movement intervention might benefit elderly individuals with early dementia remains unclear. This study takes a holistic approach to provide a detailed account of the implementation and evaluation of a dance movement intervention, to extend our understanding of the multifaceted effects of holistic health among elderly dementia patients. This RCT will be one of the first investigations to systematically compare the effects of exercise and dance movement on early dementia. With a systematic experimental design that uses longitudinal follow up and a larger sample size, paired with an evidence-based intervention, this RCT aims to confirm previous findings and provide new insights into how dance movement can provide unique benefits to elderly dementia patients. Additionally, the use of salivary cortisol will elucidate the mind-body association and provide empirical evidence of the effects of dance-movement interventions. The findings of this study will not only support the effectiveness of a dance movement based intervention, but will also provide practice models and encourage the similar development of structural programs providing elderly populations with early psychosocial support.
